# Assessing the determinants of healthcare expenditures in single-person households

**DOI:** 10.1186/s13584-018-0246-8

**Published:** 2018-10-15

**Authors:** Aviad Tur-Sinai, Racheli Magnezi, Haya Grinvald-Fogel

**Affiliations:** 1 Department of Health Systems Management, The Max Stern Yezreel Valley College, Yezreel Valley, Israel; 2 Department of Management, Faculty of Social Sciences, Bar-Ilan University, Ramat Gan, Israel; 3 Department of Management, Faculty of Social Sciences, Bar-Ilan University, Ramat Gan, Israel

**Keywords:** Healthcare expenditure, Health self-assessment, Socioeconomic level, Single-person, Social survey, Propensity score matching

## Abstract

**Background:**

The study documents a direct relationship between individuals’ health and patterns of healthcare expenditure by isolating single-person households and creating a new reference group in which household healthcare expenditure is based on one person’s expenditure patterns in accordance with his or her own state of health.

**Method:**

The study matched two surveys using Propensity Score Matching based on single-person household, age, and gender. Structural Equation Modeling (SEM) explores paths of relation between the population’s income and socioeconomic level and its health self-assessment and expenditure.

**Results:**

Single-person households’ health expenditure increases with age and the differences in most expenditure categories are significant. The current study looks into the direct and indirect effects of income, gender, and SES on health insurance and other out-of-pocket health expenses among single-person households. A direct link exists between income, gender, and socioeconomic status (SES) and several aspects of health expenditure, depending on the specific age group. The indirect effects are attested via health status assessment, in which a negative correlation is found between self-assessed health status and various health-expenditure categories.

**Conclusions:**

The last-mentioned result may support the general perception that single-person households who feel that they are doing better than their near-equals enjoy better health. This line of inquiry yields a better examination of how a single-person household’s state of health affects expenditure patterns without assuming ab initio that expenditure patterns attest to state of health.

## Background

Approximately 20.7% of households in Israel are composed of one person (Israel [6]). Presumably, the main economic decisions and ability to bear the economic burden depend on the head of household’s income [3]. Given the upward trend in the share of private funding in national healthcare expenditures [23], it may be surmised that basing healthcare on a large proportion of private funding may force many to forgo necessary medical treatment due to its cost [37].

The main costs that are privately funded are direct payments or charges for consultations with specialists, medical procedures, medicines, and tests [40]. Some regard this method of funding as representative of market failures in healthcare because they consider it inefficient and unequal [4].

In 2005, the World Health Organization, in its Resolution 58.33, affirmed the right of every person to health services regardless of his or her economic situation. Notwithstanding the resolution, the world is still very far from fulfilling the vision of universal coverage [38]. This is attributed, among other factors, to the many countries that rely on private funding to cover large shares of healthcare costs [21].

Although income is not the only factor that affects medical coverage, various population groups, such as migrants and ethnic minorities, are known to use medical services less than do other populations [32].

Furthermore, a change in people’s state of health may result in loss of income not only for them but also for relatives who take care of them. In most countries, relatives can provide some form of financial support to family members at times of illness, but formal institutional support for those who cannot continue working due to illness is less available. According to the International Labor Organization, only one-fifth of the world’s population has social insurance extensive enough to include loss of earning ability due to illness and more than half of the world’s population has no formal social-insurance protection whatsoever [38].

There is also a clear connection between a person’s socioeconomic situation and his or her state of health [7]. Abundant professional literature and copious findings on this issue demonstrate a relation between income inequality and disparities and mortality and other health indicators [1, 30, 36]. A study encompassing data from twenty-two European countries, for example, found that in almost all countries surveyed, low-socioeconomic-status groups had higher mortality rates and lower health self-assessments than those of high socioeconomic status [31]. However, the disparities among groups are smaller in West European countries than in those of Eastern Europe [17].

Income inequality affects health disparities more strongly in “neoliberal” countries than in states that are considered “social democratic,” which typically maintain social safety nets and base their health services on public funding [10]. In yet another study, however, it was concluded that support for the hypothesis that income inequality leads to health inequality in affluent countries (among them and within them) is lacking. That the strongest evidence for the existence of such a relation was found in the United States [26] may support the hypothesis that income inequality has a stronger effect on health in neoliberal countries, in which healthcare systems are typically based on private funding [16].

The international professional literature presumes that the basic unit of monetary expenditure is the household and not the individual, given that each individual’s welfare is related to the income of all household members [28]. This assumption is problematic because it disregards the implications of the state of health of an individual in a single-person household on patterns of personal expenditures including those on healthcare. Furthermore, the use of household data as the relevant expenditure unit may skew the findings because the contribution of other (healthy) household members to the coverage of expenditure makes it difficult to establish a direct relation between an individual’s state of health and its effect on spending patterns.

The current study focuses on this issue through the prism of single-person households and seeks a connection between state of health and patterns of monetary expenditure on health services among members of this population group. Examined as a subtopic are people’s subjective assessments of their state of health and the relation between these assessments and people’s patterns of financial expenditure on health services as a dependency of socio-demographic and economic indicators. The study is a milestone in the discussion of the characteristics of state of health and healthcare expenditure by single-person households—a subpopulation about which little has been done and learned so far.

## Methods

### Data

The data for the study were obtained from the Social Survey and the Household Expenditure Survey, both conducted by the Israel Central Bureau of Statistics in 2006 and 2010. The Social Survey, performed on the basis of a new sample of 9500 people each year, provides up-to-date information about the living conditions and welfare of Israel’s population, including state of health. For example, respondents are asked to judge their state of health subjectively and to define it as usually “very good,” “good,” “not so good,” or “not good at all.” The survey relates to the entire permanent population of Israel aged twenty and over.

The Household Expenditure Survey yields information about various kinds of healthcare expenditures: insurance, dental care, services such as private medical care, alternative medicine, inpatient care, psychological treatments, and other health-related expenditures such as on medicines and vitamins. It also gathers data on respondents’ income from various sources, including employment, pensions and social-insurance funds, and social benefits and assistance. Another variable used in the analysis is SES – socioeconomic status of place of residence. This variable appears on a scale from 1 ()low to 5 (high). The survey covers the entire population of households in Israel and its unit of investigation is the household, defined as a group of people who live together most days of the week and have a shared food budget. The sample comprises 6000 households each year.

### Statistical strategy

To unite the two surveys, the research population in this study was comprised of single-person households only. The two sources of information were paired using the Propensity Score Matching method, which assures a maximum fit of the characteristics of single-person households that participated in the Household Expenditure Survey with those estimated in the Social Survey. (For elaboration on the methodology, see [12].) The respondents’ gender and age were used to match the two surveys. These two variables were chosen since the information was available in both surveys. The propensity scores divided the participants into fourteen groups: men and women in seven age groups (20–24; 25–34; 35–44; 45–54; 55–64; 65–74; 75 and older). The sample size in each group varied from sixty-three men aged 20–24 to 412 women aged 75 and older.

After completing this stage, we divided the participants from the various groups into three age groups patterned after the conventional cohorts in the medical literature on the topic: young [20–29], middle-aged (30–64), and elder (65+) (see [20, 22, 41]).

For each group in the Social Survey, the mean score of the health self-assessment was calculated and matched to the equivalent group in the Household Expenditure Survey.

### Statistical analysis

Statistical analyses were performed using the SPSS statistical software, Version 25. One-way ANOVA analyses were carried out to test differences in the research variables by age groups. Pearson correlations were used to test for correlations among the study variables. The AMOS Structure Equation Modeling (SEM) program, Version 25, was run to create a path analysis with the maximum-likelihood method. SEM was used to explore paths of relation between the population’s income and socioeconomic level and its health self-assessment and expenditure.

## Results

Table 1 parses all the research variables by age groups of single-person households. Among all specific types of health expenditure, health-insurance spending is the highest, followed by dental-care expenditure and health-services expenses. All other health-related expenditure accounts for about 37% of total healthcare expenditure among households. After the data were weighted to correspond to the proportions of the age groups in the population, it was found that healthcare expenditures increased with age and that the differences in most expenditure categories were significant. Total income was highest in the middle-age group (30–64) and lowest among young participants [20–29]. Perceived health decreased significantly with age.

**Table 1:** Descriptive statistics of research variables for single-person households, by age group

Variables	20–29 M (SD)	30–64 M (SD)	65+ M (SD)	Total M (SD)
Health insurance*** (NIS)	31.0 (43.8)	134.5 (162.3)	183.5 (184.1)	142.6 (169.9)
Dental treatment*** (NIS)	68.2 (206.9)	125.0 (392.7)	152.6 (420.3)	130.3 (389.5)
Health services (NIS)	65.4 (206.4)	134.5 (593.3)	124.5 (466.7)	121.7 (505.5)
Other health-related expenditures *** (NIS)	92.9 (140.3)	188.1 (366.1)	295.9 (376.2)	229.8 (360.5)
Total income*** (NIS)	5773.2 (6168.8)	10,461.5 (9250.9)	7682.8 (19,261.9)	8629.4 (14,445.3)
Health self-assessment***	3.7 (.1)	3.1 (.4)	2.2 (.2)	2.7 (.6)
Age	24.8 (2.9)	47.5 (11.1)	76.6 (6.8)	57.4 (20.4)
Socioeconomic level***				
1-low	3.8%	2.6%	2.9%	2.9%
2	14.6%	13.2%	16.5%	14.9%
3	24.7%	28.1%	32.5%	29.6%
4	56.0%	55.7%	47.0%	51.9%
5-high	0.9%	0.3%	1.0%	0.7%
Gender**				
Male	52.7%	54.4%	21.6%	39.7%
Female	47.3%	45.6%	78.4%	60.3%
N	285	996	955	2236
N (weighted)	99,581	332,412	342,902	774,895

***P < 0.001; **P < 0.01

Table 2 shows the Pearson correlations among all research variables for single-person households. Significant positive correlations between income and health expenditure on insurance were found. Perceived health and expenditure were negatively correlated. The better the participant felt about his or her health, the less he or she spent on it.

**Table 2:** Pearson correlations among the study variables

Expenditures	Health Insurance	Dental treatment	Health services	Other health- related expenditures	Health self-assessment	Socioeconomic level	Total income
Health Insurance	1	.019	.058^*^	.158^**^	−.273**	.118***	.127**
Dental treatment	.019	1	.013	.067^*^	−.071*	−.001	.036
Health services	.058^*^	.013	1	.080^**^	−.040	.053	.043
Other health- related expenditures	.158^**^	.067^*^	.080^**^	1	−.194**	.053	.015
Health self-assessment	−.273^**^	−.071^*^	−.040	−.194^**^	1	.113***	.023
Socioeconomic level	.118***	−.001	.053	.053*	.113**	1	.126**
Income-total	.127^**^	.036	.043	.015	.023	.126***	1

***P < 0.001; **P < 0.01; *P < 0.05

**Fig. 1:**
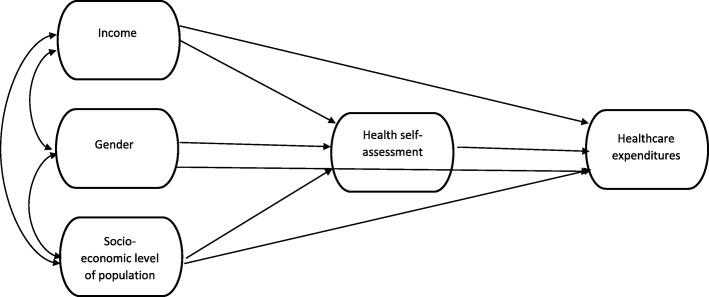
Conceptual model

The conceptual model presented in Fig. 1 suggests a correlation between single-person households’ income, socioeconomic level and gender. These are the three predictors of health self-assessment. All four variables are predictors to healthcare expenditure.

Given that significant differences by age were found (Table 1), three models were calculated for the three single-person-household age groups – young, middle, and old. All three calculations began with the full theoretical model and then reduced the variables to obtain an optimal model. Structural Equation Modeling (SEM) was carried out separately for each age group to examine predictors of healthcare expenditure and health self-assessment. In all the models, only fit indicators and significant standardized path coefficients are presented. Estimates of standardized regression weights (beta) are presented on the straight arrows in the models and correlations are shown on the curved arrows (Pearson’s coefficients- r).

**Fig. 2:**
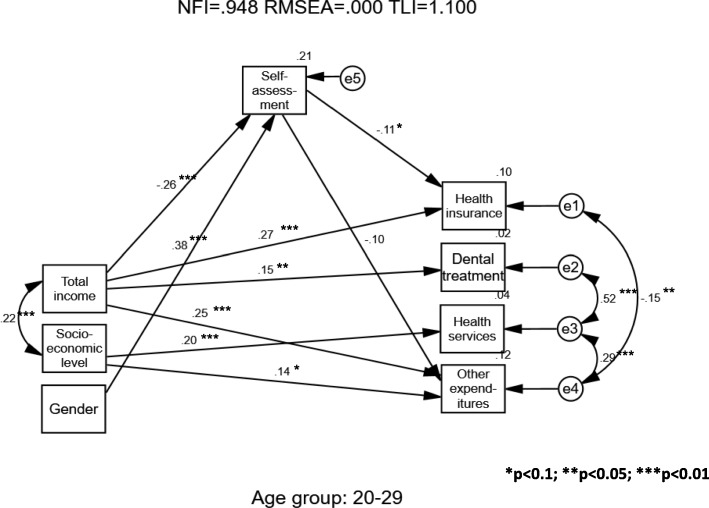
Single-Person Households – Young Sample (Age 20–29)

As may be seen in Fig. 2, the model was found to fit well among young single-person households (age 20–29) (Chi-square = 9.175 [15 df]; *p* = 0.868; CFI = 1.000; NFI = 0.948; RMSEA = 0.000; TLI = 1.100). Gender significantly predicted health self-assessment (β = 0.38), suggesting that men reported better health but did not predict any healthcare expenditures. The income significantly predicted health insurance, dental treatment, and other expenditures (β = 0.27; 0.15; 0.25 respectively). The income also negatively predicted health self-assessment (β = − 0.26).

Socioeconomic level predicted both health services and other expenditures (β = 0.20; 0.14). Health self-assessment negatively predicted health insurance and other expenditures (β = − 0.11; − 0.10). Positive significant correlations were found among dental treatment, health services, and other expenditures.

**Fig. 3:**
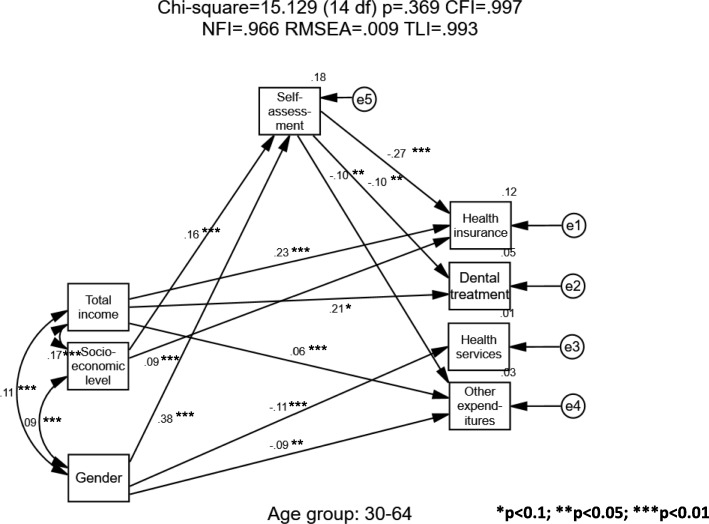
Single-Person Households – Middle-Age Sample (Age 30–64)

In Fig. 3, the model for single-person households aged 30–64 was found to fit well (Chi-square = 15.129 (14 df); *p* = .369; CFI = 0.997; NFI = 0.966; RMSEA = 0.009; TLI = 0.993). Men tended to spend less on health services and other expenditures (β = − 0.11; − 0.09), than women did, and they assessed their health more favorably (β = 0.38). The income significantly predicted insurance, dental treatment, and other expenditures (β = 0.23; 0.21; 0.06 respectively) but did not predict health self-assessment. Socioeconomic level predicted health insurance expenditure (β = 0.09) and health self-assessment (β = 0.16). Health self-assessment negatively and significantly predicted health insurance, dental treatment, and other expenditures (β = − 0.27; − 0.10; − 0.10, respectively).

**Fig. 4:**
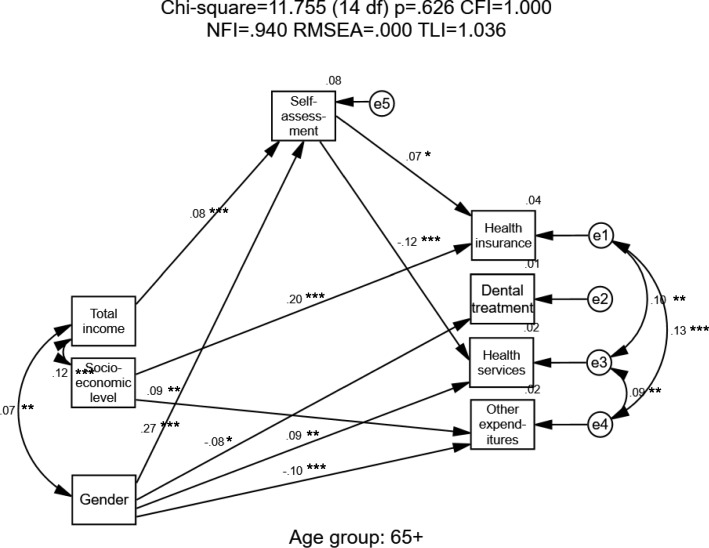
Single-Person Households – Old-Age Sample (Age 65+)

In Fig. 4, for single-person households aged 65+, a good fit (Chi-square = 11.755 (14 df); *p* = .626; CFI = 1.000; NFI = 0.940; RMSEA = 0.000; TLI = 1.036) was found for the model. Men tended to spend less on dental treatment and other expenditures but more on health services (β = − 0.08; − 0.10; 0.09, respectively); they also assessed their health more positively than did women (β = 0.27). Higher single-person households’ socioeconomic levels directly predicted higher expenditure on health insurance and other expenditures (β = 0.20; 0.09). Income significantly predicted health self-assessments only but none of the healthcare expenditures. Health self-assessment positively predicted health insurance expenditures (β = 0.07) and negatively predicted health services expenditures (β = − 0.12), suggesting that people who perceive their health positively spend more on health insurance and less on health services.

## Discussion

When illness strikes, many people may incur an economic burden or even exhaust their resources as lack of treatment for a medical condition deprives them of income-earning ability. Many who seek medical care also encounter economic difficulties due to attendant out-of-pocket costs. In this context, it is known that the ability to obtain needed medical services depends to some extent on the income level of an individual or a household.

In Israel, the share of private funding of medical services has risen to 39% of all healthcare-system expenditure over the past decade [8]. This is thought to be the highest proportion of such funding among developed countries that offer universal health insurance [23]. As a corollary, it has been argued that this trend has an immediate limiting effect on access to health services and on households’ ability to afford them [9].

This study used single-person households as a new approach toward referencing and investigated factors that affect healthcare expenditure and perceived state of health. Given that health is usually a “normal good,” one would expect to find a direct relation between increased income and health and its derivatives. Many variables, however, may impact this delicate balance [5]. For the current study, the focus was placed on whether household structure may be categorized as a disruptor of this balance.

Single-person households were found to spend more on health insurance than on other specific kinds of health expenditure. This result reinforces findings on the growing prevalence of households’ use of and dependence on health services that are not necessarily provided by the public sector and, hence, their need to obtain additional medical protection, as permitted by the Israel National Health Insurance Law [13], by purchasing supplementary insurance.

Health expenditure among single-person households increases with age and does so significantly in most expenditure categories. These results reinforce previous research on this topic [33, 34]. Congruent with previous findings [14, 15], we found that perceived health decreases significantly with age, the obvious explanation being that the more people age, the more health problems they have.

The current study looked for the first time at the direct and indirect effects of income, gender, and SES on health-insurance and other out-of-pocket health expenditure among single-person households. The indirect effects were gauged through the proxy of health status assessment. The study attempted to document a direct relation between an individual’s state of health and his or her patterns of healthcare expenditure by isolating single-person households and creating a new reference group in which household healthcare expenditure is based on one person’s expenditure patterns in accordance with his or her own state of health. The study also augments previous research by cross-referencing data on single-person-household expenditure patterns with data attesting to the way single-person households assess their state of health. As we pursued the inquiry, we assumed that healthcare-expenditure patterns reflect the existence or nonexistence of a health problem in the single-person household and that a direct connection exists between single-person households’ self-assessed state of health and their healthcare spending patterns. This line of inquiry allowed us to examine the way state of health affects expenditure patterns without assuming ab initio that expenditure patterns attest to state of health.

We began by dealing with the direct effects of gender, income, and SES on health expenditure. On this basis, we extended our discussion to the indirect effect via health status assessment.

Gender was found to have no significant effect on healthcare expenditure among single-person households aged 20–29. This result is unique to this age group. Among adult (age 30–64) single-person households, men tend to spend less on health services and on other kinds of health expenditure. No gender disparity, however, was found among adult single-person households in patterns of health-insurance and dental-treatment expenditure. Similar results have been found among adults in the U.S. [2, 24]. Conversely, in Italy women’s healthcare expenditures were significantly lower than men’s [11]. The results in regard to elder (65+) single-person households yielded a mixed picture between that obtained for young single-person households and that found among adult single-person households. As was found among adult single-person households, a difference exists between households headed by men and those headed by women in regard to the correlation with the patterns of health expenditure. Namely, among older single-person households, men tend to spend less on dental-health treatments and other health expenditures and more on health services. In patterns of expenditure on health insurance, however, no gender disparity among adult single-member households was found. Further research should explain the reasons for the differences between Israel and Europe among the age groups and determine whether these differences recur in non-single-person households.

As for the relation between gender and self-assessed state of health, it was found that male single persons assess their state of health more positively than do female single persons, irrespective of age. This may be attributable to individuals’ sources of income and level of wealth, and, by extension, their ability to manage their health in a way that would assure them a satisfactory and adequate level of health.

While income and self-assessed state of health were found to be negatively related among people who live alone in early age, among elders who live on their own this relation was found positive. This may be explained in the way households relate to “unattainable income” [18]. In other words, single-person households have a single source of income by definition, whereas elder single-person households’ income level has been trending upward over the years and is positively correlated with self-assessment of state of health.

The study revealed a positive relation between the income level of a young single-person household and its level of expenditure on health insurance, dental care, and other health expenditure. The relation between income level and healthcare-services expenditure among adult single-person households resembles that obtained in regard to young single-person households [39].

Research on non-single-person households in the same respects shows that income usually has a modest but positive impact on health-insurance expenditure [25]. Although we found no previous studies regarding this behavior among single-person households, the following extrapolation from Theodossiou and Zangelidis [35] is plausible: At this relatively young age, “social status” is not yet completely defined; thus, the real connection between health and income without the influence of social perception is encountered. To connect these results with the current study, one may consult a large study in Spain showing that single persons report higher self-perceptions of health than do people in other categories. As for gender, a difference between men and women is found only in specific kinds of single-person households. Taking single persons as the reference category, only men separated from their wives had significantly lower levels of self-reported health; widows and divorcées were more likely to perceive their health as worse than that of single women [5].

In contrast to the findings among young and adult single-person households in regard to the relation between income level and health-services expenditure, no relation whatsoever between these aspects was found among elder single-person households. The explanation for this may have to do with the possibility that elder single-person households consider health expenditure a “must” for the assurance of their health; consequently, a change in their income level does not find expression in their patterns of expenditure on healthcare services.

As for the patterns of the relation between a single-person household’s socioeconomic level and its expenditure on health, however, a positive relation is found, much as among young single-person households, between elder single-person households and other health expenditure. Furthermore, much as in the case of elder single-person households, a positive relation is found between the elder single-person household’s socioeconomic level and its expenditure on health insurance.

The general picture that emerges shows that single-person households show a positive relation between socioeconomic level of place of residence and expenditure on health and other services. Although this outcome seems expectable [19], variance is found in the structure of the relation between these two elements as a function of household age. Namely, among young households the relation is positive in regard to health-services expenditure and other health expenditure. Among adult households, it is positive for dental-care expenditure only, whereas among elder households it is positive in regard to dental care and other health expenditure. In addition, socioeconomic level was found to be predictive of health self-assessment. The fact that socioeconomic status of place of residence directly explains some expenditure patterns of single-person households and has no predictive power whatsoever in regard to health self-assessment (with the exception of adult households) shows that this motive should be seen as a homogeneous dimension among all single-person households in a given area of residence.

We now deal with the indirect effects of health status assessment on health-insurance and other out-of-pocket health expenditures. A negative correlation is found between the self-assessed state of health of a person living alone at an early age and his or her expenditure on health insurance. A similar finding is obtained in regard to the pattern of the relation between self-assessed state of health of a young individual living alone and other health expenditure. This outcome corroborates previous results on the patterns of the relation between individuals’ subjective assessment of state of health and their healthcare expenditure patterns [27, 29]. It also underscores the concern about exacerbating inequality in the healthcare system due to the need to assure funding of healthcare expenditure for single-person households, whose sources of income are only their own, in contrast to households that have multiple breadwinners.

The adult single-person household’s self-assessed state of health is found negatively correlated not only with health-insurance expenditure—as is also found among young single-person households—but also with expenditure on dental health and other health expenditure. The explanation for this may trace to the absolute level of expenditure on these two items, which foists a severe burden on total income of the adult single-person household—requiring its head to examine with greater concern and stringency the need to fund this expenditure.

Single-person households headed by elder men assess their state of health more positively than do single-person household headed by elder women. This result, as stated, is consistent with the outcomes obtained in regard to young or adult single-person households, as described above. A possible explanation for men’s advantage over women in assessing their state of health is that this outcome may be attributed to the question of individuals’ sources of income and level of wealth and, by extension, their ability to manage their health in a way that would assure them a satisfactory and adequate level of health.

The self-assessed state of health of an elder single-person household is positively correlated with health-insurance expenditure and negatively correlated with health-services expenditure. The positive correlation between the self-assessed state of health of a single-person household headed by an elder and health-insurance expenditure may be seen as an oblique acknowledgment by elder individuals of the need to make this expenditure for their financial protection in later years—an expenditure that they consider worth the money. Where other medical expenditures are concerned, however, the perception of a single-person household headed by an elder is the opposite: it is considered a tax-like expense that may not be worth its while. Therefore, the correlation found is negative.

The current study has several noteworthy limitations. Given the need to identify single-person households and to match information concerning state of health and that on consumption of healthcare services, we used the propensity score matching method, which we find statistically the most optimal way to proceed on the basis of the existing stock of statistical data. Due to the structure of the surveys, however, we could not generalize certain kinds of information, such as participants’ level of education, patterns of health behavior, and patterns of employment.

To improve future research on these issues, we believe, some improvement in data collection is warranted. As this is done, ethnic background, education level, practices such as smoking and exercise, occupation, and history of specific illnesses should be among the relevant estimated variables that can be harvested from the relevant databases.

The current study investigated single-person households at one time only. Future research should broaden the discussion by asking whether these households’ patterns of health and consumption of health services change over the years and whether different groups of households have weaknesses in their behavior patterns.

## Conclusions

The current study focuses on the direct and indirect effects of individuals’ socioeconomic and health characteristics on spending patterns. The study is a milestone in the discussion of the characteristics of state of health and healthcare expenditure among single-person households—a sub-population about which little has been done and learned so far.

Several important points come to light in regard to the matrix of relations and patterns that predict health expenditure among single-person households. First, it is found that the self-assessed state of health of a male-headed single-person household is higher than that of a woman who lives alone, irrespective of age. Second, the subjective assessment of state of health among single-person households is a mediating variable on the question of the relation between a household’s exogenous and socioeconomic characteristics and its patterns of health expenditure. It is also found that among adult and elder single-person households, male heads of household usually spend less on various kinds of health services than do female ones in the same age group. Furthermore, while a positive relation exists between the income level of a young or an adult single-person household and the level of expenditure on various kinds of health services, these two aspects are wholly unrelated among elder single-person households. The reason may trace to the possibility that these households consider health expenditure a “must” for the assurance of their health; meaning that a change in their income level does not find expression in their patterns of expenditure on health services.

The present study augments the existing theoretical literature by specifically investigating and estimating the relationship between state of health and patterns of healthcare expenditure among single-person households. In further research, we plan to compare the extent of the decrease in perceived health between single- and non-single-person households, excluding covariates such as socioeconomic status, prior health, etc. [14].

Given the large proportion of single-person households in Israel as well as the national demographic forecast, the healthcare system and policymakers will have to be more mindful of the profile of single-person households than they have been and should ensure that their in-house analysts or outside researchers examine these households’ patterns of healthcare expenditure as a function of age.

Furthermore, in view of the permanent upward trend in healthcare-service prices, Israel’s healthcare system and policymakers should examine alternative ways of restraining elder households’ expenditure on dental care. This might be done, for example, by increasing the supply of dental-care services delivered by health funds (HMOs) for elder population groups and by broadening the set of subsidized dental-care services for these populations.

Policymakers should also seek alternative sources of funding for these households’ health-insurance outlays. One way of doing this would be to expand the set of medical services that the Ministry of Health and the HMOs provide for these population groups. Another possibility is to lower these patients’ out-of-pocket expenditure on health coverage exceeding that provided at the public level and by the HMOs.
